# Predictive value of EGSYS score in the differential diagnosis of cardiac syncope and neurally mediated syncope in children

**DOI:** 10.3389/fcvm.2023.1091778

**Published:** 2023-03-17

**Authors:** Yunxi Li, Jianglin Liu, Minmin Wang, Haizhao Zhao, Xiaoyue Liu, Jing Hu, Cuifen Zhao, Qingyu Kong

**Affiliations:** ^1^ Department of Pediatrics, Qilu Hospital, Cheeloo College of Medicine, Shandong University, Jinan, China; ^2^ Department of Pediatrics, Children's Hospital Affiliated Shandong University, Jinan, China

**Keywords:** syncope, cardiac syncope, neurally mediated syncope, children, EGSYS

## Abstract

**Background and objective:**

Syncope is a common emergency with diverse etiologies in children. Among these, cardiac syncope (CS) is associated with high mortality and is usually difficult to diagnose. However, there is still no validated clinical prediction model to distinguish CS from other forms of pediatric syncope. The Evaluation of Guidelines in Syncope Study (EGSYS) score was designed to identify CS in adults and has been validated in several studies. In this study, we aimed to assess the ability of the EGSYS score in predicting CS in children.

**Methods:**

In this retrospective study, we calculated and analyzed the EGSYS scores of 332 children hospitalized for syncope between January 2009 and December 2021. Among them, 281 were diagnosed with neurally mediated syncope (NMS) through the head-up tilt test, and 51 were diagnosed with CS using electrocardiography (ECG), echocardiography (ECHO), coronary computed tomography angiography (CTA), myocardial enzymes and genetic screening. The receiver operating characteristic (ROC) curve and Hosmer-Lemeshow test were used to evaluate the predictive value of the EGSYS score system.

**Results:**

The median scores of 51 children with CS and 281 children with NMS were 4 [interquartile range (IQR): 3-5] and −1 (IQR: -2-1), respectively. The area under the ROC curve (AUC) was 0.922 [95% confidence interval (CI): 0.892-0.952; *P *< 0.001], indicating that the EGSYS score system has good discrimination. The best cutoff point was ≥3, with a sensitivity and specificity of 84.3% and 87.9%, respectively. The Hosmer-Lemeshow test demonstrated satisfactory calibration (*χ*²=1.468, *P *> 0.05) of the score, indicating a good fit of the model.

**Conclusion:**

The EGSYS score appeared to be sensitive for differentiating CS from NMS in children. It might be used as an additional diagnostic tool to aid pediatricians in accurately identifying children with CS in the clinical practice.

## 1. Introduction

Syncope is a transient loss of consciousness (TLOC) and postural tone due to cerebral hypoperfusion followed by rapid and spontaneous complete recovery. Syncope is estimated to occur in approximately 50% of the general population during their lifetime (1). As one of the most common emergencies, syncope accounts for approximately 1% of pediatric emergency department visits (2). There are three major classifications of syncope in children and adolescence: neurally mediated syncope (NMS), cardiac syncope (CS), and unexplained syncope. NMS includes vasovagal syncope (VVS), postural tachycardia syndrome (POTS), orthostatic hypotension (OH), and orthostatic hypertension (OHT). NMS are mostly benign with the main threats leading to syncope-related body injuries or psychological problems (3, 4). However, CS may be associated with a high risk of sudden cardiac death or other adverse events especially in those caused by life-threatening arrhythmia or structural cardiac disease (5–7). Therefore, children with CS require early diagnosis and urgent disease-specific therapy to improve their prognosis.

Nearly 50% of cases can be diagnosed using a detailed patient history, physical examination, and electrocardiography (ECG) (8). However, several studies have shown that the hospital admission rate and medical costs remain high for patients with syncope due to concerns about underlying cardiac disease (9, 10). On the other hand, children with CS especially those caused by a malignant arrhythmia, may have no distinctive abnormal manifestations after syncopal episodes resulting in difficulties in timely diagnosis.

To help triage and manage patients with syncope in the emergency department, Del Rosso et al. (11) developed the Evaluation of Guidelines in Syncope Study (EGSYS) score which is a clinical risk-scoring system to differentiate CS from non-CS. Although this model has been validated in several studies in adults (12–16), whether or not the EGSYS score can be used to identify CS in children is unknown. A recent study by Környei et al. (17) showed that the EGSYS score identified cardiac causes in seven of eight arrhythmic patients with syncope, suggesting the usefulness of the EGSYS score in children with syncope of arrhythmic origin. However, this score has not been tested in other types of pediatric syncope.

In this study, we calculated and analyzed the EGSYS scores of 332 children with syncope of different causes and assessed the predictive value of EGSYS score for the differential diagnosis between CS and NMS in children.

## 2. Materials and methods

### 2.1. Subjects

This retrospective observational cohort study was conducted at a large tertiary hospital, Qilu Hospital of Shandong University, between January 2009 and December 2021. We reviewed the clinical data of all children with syncope obtained from our hospital's electronic information system. Subsequently, we included those (a) between the ages of 0 and 18; and (b) diagnosed with NMS and CS, and excluded those (a) with unexplained syncope; (b) incomplete or missing crucial medical records; and (c) TLOC caused by epilepsy, hypoglycemia, hyperventilation, intoxication, hypoxia, transient ischemic attacks, or psychological factors. Finally, 332 children with syncope were included in this study. The diagnostic criteria for NMS and CS were based on the Chinese Pediatric Cardiology Society (CPCS) guidelines for the diagnosis and treatment of syncope (18–20) and European Society of Cardiology (ESC) guidelines for the diagnosis and management of syncope (1, 21).

The data were collected based on the medical records during hospitalizations and included the demographic data (age, sex), medical history, physical examination; the results of 12-lead ECG, 24 hour Holter monitor, and echocardiograph (ECHO). A detailed medical history included the predisposing factors, prodromes, duration of loss of consciousness, family history, and past medical history. Finally, we calculated and analyzed the EGSYS score of each child to explore its predictive value for the differential diagnosis between CS and NMS in children.

Informed consent was obtained from all the children in this study, and the study was approved by the ethics committee of Qilu Hospital, Shandong University (approval No. KYLL-202210-033).

### 2.2. Calculation of EGSYS score

The EGSYS score consists of six predictors (11, 13) (show in Table 1): (1) Abnormal ECG and/or structural heart disease (plus three points), (2) palpitations preceding syncope (plus four points), (3) syncope during effort (plus three points), (4) syncope while supine (plus two points), (5) precipitating and/or predisposing factors (minus one point), and (6) autonomic neurovegetative prodromes (minus one point)*.* All predictors were evaluated for each patient by two trained pediatricians. Then, a total score ranging from −2 to 12 points was calculated as the sum of the points assigned to each predictor. Finally, each patient's score was determined after review by pediatric cardiovascular experts.

**Table 1:** The evaluation of guidelines in syncope study (EGSYS) scores^a^.

Predictors	Point
Abnormal ECG and/or structural heart disease^b^,^c^	+3
Palpitations	+4
Syncope during effort	+3
Syncope in supine position	+2
Precipitating and/or predisposing factors^d^	−1
Autonomic neurovegetative prodromes^e^	−1

^a^ Adapted from Del Rosso et al (11).and Kariman et al (14). A score ≥3 indicates a high risk of cardiac syncope.

^b^ ECG abnormality was defined as any of the following: sinus bradycardia lasting <40 min, sinus pauses lasting >3 s, ST changes (elevation or depression) > 1 mm, alternating left and right bundle branch block, Mobitz II second- and third- degree atrioventricular block, sick sinus syndrome, QT prolongation ≥440 ms, ventricular tachycardia or paroxysmal supraventricular tachycardia, and pacemaker malfunction.

^c^ structural heart disease was defined as having any of the following: congestive heart failure, ischemic disease, valvular dysfunction, cardiomyopathy, and congenital heart disease.

^d^ precipitating and/or predisposing factors included one or more of the following conditions: a change in posture, during or after exercise, during or immediately after urination, defecation, cough, swallowing, crowded or warm places, prolonged standing, postprandial period, fear, intense pain, and neck movements.

^e^ Neurovegetative prodromes included the following: blurred vision, dizziness, nausea, vomiting, abdominal discomfort, feeling cold or warm, tremors, sweating, and pallor.

### 2.3. Statistical analysis

All statistical analyses were performed using SPSS version 25.00 (IBM Corporation, New York, United States) and GraphPad Prism version 8.0. Non-normally distributed continuous data were described as median (interquartiles: P25, P75) and differences between groups were compared using the Mann-Whitney *U* test. Qualitative data were expressed as numbers and percentages (%), and comparisons between the two groups were performed using chi-square tests. The ROC curve was adopted to analyze the discrimination of the EGSYS score. The area under the curve (AUC) represented how well the EGSYS score differentiated cardiac syncope with values of 0.5–0.7, 0.7–0.9, and > 0.9 indicating a low, moderate and high diagnostic value respectively. The best EGSYS score cutoff value was determined based on the maximum Youden index value. The Hosmer-Lemeshow test was used to assess the goodness of fit of the EGSYS score model. A *P* > 0.05 suggested there was no statistically significant between predictive values and observed values, that is, an acceptable fit of the EGSYS score. Statistical significance was set at *P* < 0.05.

## 3. Results

### 3.1. Demographic features

A total of 332 children were included in the study (Table 2). Of these, 281 children (130 males and 151 females) with a median age of 11.4 (9.7,12.9) years were diagnosed with NMS and 51 children (26 males and 25 females) with a median age of 13.0 (10.7,15.0) years were diagnosed with CS. There were no statistical differences in age or sex between the two groups (*P* > 0.05).

**Table 2:** Demographic and clinical features of children included.

Variables, *n* (%)	Cardiac syncope (*N* = 51)	Neurally mediated syncope (*N* = 281)	*P*
Onset of age, M(IQR:P25 P75) (years)	13.0 (10.7,15.0)	11.4 (9.7,12.9)	0.075
Gender, *n* (M/F)	26/25	130/151	0.535
Syncope frequency, *n* (%)
1	22 (43.1%)	72 (25.6%)	0.025
2–5	25 (49.0%)	166 (59.1%)	
>5	4 (7.8%)	43 (15.3%)	
Clinical course, *n* (%)
≤1 month	21 (41.2%)	126 (44.8%)	0.225
>1month, <1 year	13 (25.5%)	92 (32.7%)	
≥1 year	17 (33.3%)	63 (22.4%)	
Duration of loss of consciousness, *n* (%)
≤1 min	26 (51.0%)	114 (40.6%)	0.137
>1 min, <5 min	13 (25.5%)	113 (40.2%)	
≥5 min	12 (23.5%)	54 (19.2%)	
Precipitating factors, *n* (%)
During exercise	28 (54.9%)	24 (8.5%)	<0.001
After exercise	2 (3.9%)	29 (10.3%)	0.237
Prolonged standing	0	110 (39.1%)	<0.001
Change in Position	0	33 (11.7%)	<0.01
Emotional	1 (2.0%)	16 (5.7%)	0.443
Special situation	0	39 (13.9%)	0.005
Prodromes, *n* (%)
Dizziness	16 (31.4%)	141 (50.2%)	0.013
Blurred vision	9 (17.6%)	137 (48.8%)	<0.001
Diaphoresis	1 (2.0%)	69 (24.6%)	<0.001
Palpitations	8 (15.7%)	33 (11.7%)	0.431
Pallor	3 (5.9%)	92 (32.7%)	<0.001
Gastrointestinal symptoms	6 (11.8)	92 (32.7%)	0.003
Chest tightness/chest pain	15 (29.4%)	51 (18.1%)	0.064
Position, *n* (%)
Supine	3 (5.9%)	2 (0.7%)	0.027
Upright	36 (70.6%)	223 (79.4%)	0.164
Siting	8 (15.7%)	22 (7.8%)	0.125
Any body position	3 (5.9%)	2 (0.7%)	0.380
Family history of sudden death or syncope, *n* (%)
Yes	3 (5.9%)	13 (4.6%)	0.976
No	48 (94.1%)	268 (95.4%)	
History of cardiovascular diseases, *n* (%)
Yes	9 (17.6%)	5 (1.8%)	<0.001
No	42 (82.4%)	276 (98.2%)	
Abnormality in physical examination, *n* (%)	31 (60.8%)	31 (11.0%)	<0.001

### 3.2. Comparison of clinical characteristics between two groups

Table 2 also shows the clinical features of the children with NMS and CS. Significant differences in precipitating factors, prodromes, supine position, history of cardiovascular disease, and physical examination were identified (*P *< 0.05).

### 3.3. Underlying diseases of children included

All 281 children with NMS underwent a head-up tilt test. Among them, there were 176 cases of VVS, 26 cases of POTS, 75 cases of VVS with POTS, 3 cases of OH, and 1 case of OHT. In the CS group, there were 27 cases of arrhythmia, 9 cases of structural heart disease, and 15 cases with both causes. The underlying diseases in the children with CS are listed in Table 3.

**Table 3:** The underlying disease of children with cardiac syncope.

Disease	*N* (%)	Proportion
Arrhythmia	27	52.9%
Paroxysmal supraventricular tachycardia	7	13.7%
Wolff-Parkinson-White syndrome	3	5.9%
Ventricular tachycardia	1	2.0%
Mobitz II second- and third-degree atrioventricular block	6	11.7%
Sick sinus syndrome	2	3.9%
Catecholaminergic polymorphic ventricular tachycardia	4	7.8%
Congenital long QT syndrome	4	7.8%
Structural cardiac disease	9	17.6%
Valvular aortic stenosis	2	3.9%
Pulmonary stenosis	1	2.0%
Idiopathic pulmonary hypertension	3	5.9%
Aortopulmonary septal defect + pulmonary hypertension	1	2.0%
Anomalous origin of coronary artery	1	2.0%
Atrial myxoma	1	2.0%
Both causes	15	29.4%
Acute myocarditis + arrhythmia	10	19.6%
Dilated/hypertrophic cardiomyopathy + arrhythmia	5	9.8%

### 3.4. Comparison of EGSYS scores between two groups

A comparison of EGSYS risk factors between the NMS and CS groups is shown in Table 4. Children with CS were more likely to have abnormal ECG findings and/or underlying heart disease (*P *< 0.05). The rates of syncope attacks during effort and in the supine position were significantly higher in children with CS than in those with NMS (*P* < 0.05). Autonomic prodromes were more common in the NMS group than in the CS group (*P *< 0.05). The results of the EGSYS risk factors in the multivariable analysis are shown in Table 5.

**Table 4:** Comparison of EGSYS risk factors between two groups.

Variable	Cardiac syncope (*N* = 51)	Neurally mediated syncope (*N* = 281)	*χ*²/Z	*P*
Abnormal ECG and/or structural heart disease (*n*,%)	46 (90.2%)	42 (14.9%)	125.473	<0.001
Palpitations preceding syncope (*n*,%)	8 (15.7%)	33 (11.7%)	0.620	0.431
Syncope during effort (*n*,%)	28 (54.9%)	46 (16.4%)	37.000	<0.001
Syncope in supine position (*n*,%)	4 (7.8%)	2 (0.7%)	8.678	0.003
Precipitating and/or predisposing factors (*n*,%)	31 (60.8%)	200 (71.2%)	2.201	0.138
Autonomic neurovegetative prodrome (*n*,%)	24 (47.1%)	251 (89.3%)	54.221	<0.001
EGSYS score
≥3	43 (84.3%)	34 (12.1%)	−9.864	<0.001
<3	8 (15.7%)	247 (87.9%)		
M (P25, P75)	4 (3, 5)	−1 (−2, 1)		

**Table 5:** The result of EGSYS risk factors on multivariable analysis.

variable	OR	B	*P*	Score
Abnormal ECG and/or structural heart disease	74.232	4.307	<0.001	3
Palpitations preceding syncope	2.556	0.938	0.162	4
Syncope during effort	34.791	3.549	<0.001	3
Syncope while supine	9.052	2.203	0.068	2
Precipitating and/or predisposing factors	0.183	−1.689	0.013	−1
Autonomic neurovegetative prodromes	0.313	−1.160	0.035	−1

### 3.5. Predictive value of EGSYS score

**Figure 1:**
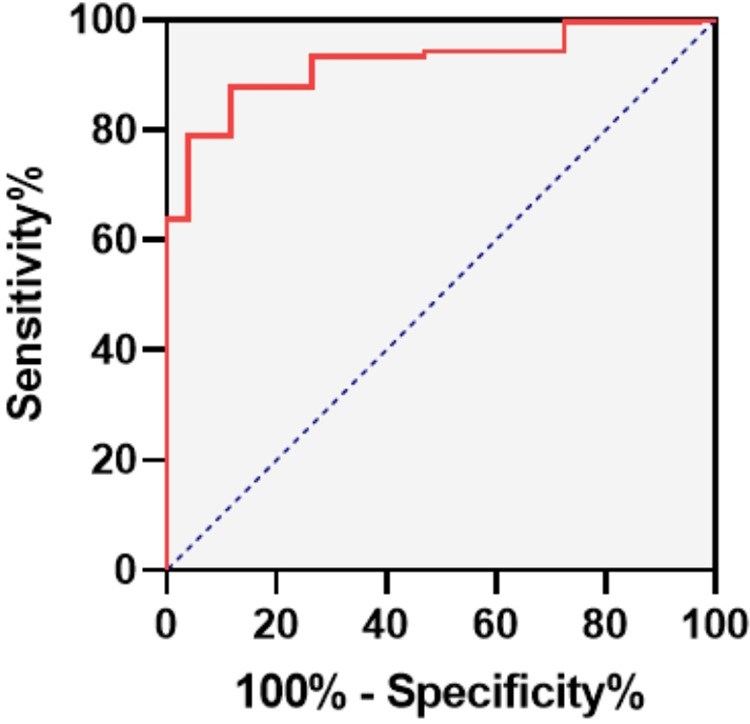
The receiver operating characteristic curve of EGSYS score.

The median EGSYS scores of the CS and NMS groups were 4 (3, 5) and −1 (-2, 1) respectively (*P *< 0.05; Table 4). The ROC curve for the EGSYS score is shown in Figure 1. The AUC of the EGSYS score was 0.922 [95% confidence interval (CI) 0.892–0.952; *P *< 0.001]. An EGSYS score ≥ 3 was the best cutoff value for diagnosing CS in children, with a sensitivity and specificity of 84.3% (95% CI 0.709–0.925) and 87.9% (95% CI 0.834–0.914) respectively. The Hosmer-Lemeshow test demonstrated good results for the goodness-of-fit of the model (*χ*^2 ^= 1.468, *P *= 0.917).

## 4. Discussion

Accurate diagnosis and risk stratification are extremely important for the proper management of patients presenting with syncope. In past researches, several syncope prediction tools based on clinical characteristics were developed and validated in adults (14, 22, 23). The prediction tools used to access the serious event in short-term include the San-Francisco Syncope Rule (SFSR), the Boston Syncope Criteria (BSC), the Risk Stratification of Syncope in the Emergency Department (ROSE) risk score and the Canadian Syncope Risk Score (CSRS) (24). The Osservatorio Epidemiologico sulla Sincope del Lazio (OESIL) risk score is to predict mortality at one year (24). However, these tools have a limited value in children. The SFSR and the ROSE require invasive laboratory tests. The CSRS incorporates a physician's diagnostic impression. The BSC and the OESIL include acquired cardiovascular risk factors, such as age, acute coronary syndrome, and heart failure. In contrast, impaired structural cardiac diseases and cardiac ion channelopathy are more common in younger patients with CS (25). The EGSYS score developed by Del Rosso et al. includes six clinical parameters: abnormal ECG and/or heart disease, palpitations, syncope during exertion, syncope in a supine position, predisposing factors and/or predisposing factors, and autonomic prodromes. Del Rosso et al. reported that the sensitivity and specificity of the EGSYS score for the diagnosis of CS were 92% and 69% respectively. Although several subsequent studies have also indicated that the EGSYS score has acceptable discrimination, it has not been validated in children with syncope.

In this study, we found that NMS and CS had different clinical characteristics in terms of predisposing factors, precursors, supine position, cardiovascular diseases, physical examination, and ECG results. After retrospectively analyzing the clinical data of 332 children with NMS and CS, we found that the calculated EGSYS scores had good sensitivity and specificity for the diagnosis of CS in children. According to the current guidelines for the management of syncope in children and adolescents (1, 26, 27), NMS is mostly triggered by prolonged standing, postural change, dehydration, urination, coughing, emotional surges, and stuffy or crowded environments. Prodromes, such as dizziness, nausea, abdominal discomfort, visual blurring, sweating, hyperventilation, and pallor appear either singularly or in combination before the onset of syncope. CS is more likely to occur during exertion or in the absence of a stimulus. Children with CS often have no prior autonomic prodrome but may sometimes present with chest tightness/pain and palpitations. Children with NMS are usually faint in an upright posture, whereas those with CS may have a syncopal event in any position. Additionally, abnormalities in the physical examination and 12-lead ECG indicated the possibility of cardiac causes. As shown in Table 1, the major risk factors of CS provided by the guidelines have been included in the EGSYS score; thus, the scoring model may help identify patients at higher risk of CS.

Although palpitations preceding syncope had the highest weight in the EGSYS score, our study showed no significant difference between the CS and NMS groups. In adults, palpitations are a common symptom of arrhythmia, such as supraventricular or ventricular tachycardia, atrial fibrillation, and atrial flutter. In such instances, palpitations are accompanied by dizziness, syncope, or near syncope (28). However, palpitations are rarely seen in pediatric clinics and emergency rooms. Children have difficulty understanding the meaning of palpitations and expressing them accurately. Zhang et al. reported a similar phenomenon (29). Therefore, palpitations may be an underrepresented feature in children with CS. However, Hurst et al. (2) analyzed CS patients (age 11.5 ± 4.5 years) in the emergency department and proposed that having palpitations was an indicator predicting CS in the pediatric emergency department, with a sensitivity of 100% and a specificity of 98%. Presently, palpitation-preceding events are still considered “red flags” of CS in many guidelines. Therefore, palpitations immediately followed by syncope should raise suspicions of CS in older children and adolescents.

This is the first study to externally validate a scoring system for predicting CS in children. Normally, an ideal predictive model properly identifies each patient who has an event from those who do not, without misclassification (30). In this study, the AUC of the EGSYS score was 0.922, demonstrating good accuracy in discriminating CS. The optimal cut-off was an EGSYS score of 3, which was consistent with the result reported by Del Rosso et al. (11). An EGSYS score ≥ 3 indicates a high possibility of CS with a sensitivity of 84.3% and a specificity of 87.9%. In such cases, more examinations such as echocardiography, 24 hour Holter monitoring, cardiac electrophysiology, and genetic screening are needed to identify the underlying disease. Furthermore, the *P*-value of 0.917 for the Hosmer-Lemeshow test represented a satisfactory calibration of the model.

## 5. Conclusion

Our study suggested that the EGSYS score appeared to be sensitive for differentiating CS from NMS in children. It might be used as an additional diagnostic tool to aid pediatricians in accurately identifying children with CS in the clinical practice. Further studies in larger populations are required to validate and modify the scoring system for clinical application.

## 6. Limitations

The study had some limitations. It was a single-center retrospective study in hospitalized children, and the number of children with CS was small. We took medical history and made the diagnosis of CS and NMS according to widely accepted CPCS guidelines and ESC guidelines. All patients' diagnoses and medical records were reviewed by pediatric cardiovascular specialist prior to discharge to minimize misclassification. Another concern is the possible recall bias. We acknowledge the undeniable fact that recall bias exists in retrospective studies. In our study, we have excluded those with incomplete and unclear information. Furthermore, the age, sex, and the time since the first syncope episode showed no statistical differences between the two groups. Thus, we think that recall bias was present to a similar extent in both groups, and it was unlikely to have affected our conclusion. In addition, we did not complete the follow-up of all children, so the predictive value of the model for prognosis is unclear. It is necessary to conduct further prospective, multi-center studies with larger sample sizes to validate and modify the EGSYS score in the diagnosis and risk stratification of CS in children. Despite these limitations, the study demonstrated that the EGSYS score might be a simple and useful predictive tool for pediatricians.

## Data Availability

The original contributions presented in the study are included in the article/Supplementary Material, further inquiries can be directed to the corresponding authors.
